# Risk factors and nomogram model for recurrence of benign paroxysmal positional vertigo in postmenopausal women: a multicenter cross-sectional study

**DOI:** 10.3389/fneur.2025.1595887

**Published:** 2025-05-12

**Authors:** Qing-Chun Pan, Bei Li, Kai Zou

**Affiliations:** Department of Otolaryngology, Head and Neck Surgery, Affiliated Hospital of North Sichuan Medical College, Nanchong, China

**Keywords:** canalith repositioning procedure, benign paroxysmal positional vertigo, postmenopausal women, estradiol, 25(OH)D, calcium, nomogram

## Abstract

**Objective:**

To explore the risk factors for recurrent benign paroxysmal positional vertigo (BPPV) in postmenopausal women within 1 year of canalith repositioning procedure (CRP), and develops a risk model based on serum 25-hydroxyvitamin D (25(OH)D), estradiol, and calcium levels to provide early identification of high-risk groups and guide prevention and treatment strategies.

**Methods:**

Data from postmenopausal women with BPPV, diagnosed and successfully treated with CRP at five hospitals in Sichuan Province between January 2019 and January 2024, were retrospectively analyzed. Participants were divided into BPPV validation and training sets in a 3:7 ratio. Clinical data were categorized into recurrence and non-recurrence subgroups based on whether BPPV recurred after treatment. LASSO regression identified factors influencing recurrence within 1 year after CRP, and multivariate logistic regression (MLR) analysis was used to develop a risk nomogram prediction model (NPM).

**Results:**

A total of 490 patients were enrolled, with 147 in the validation set and 343 in the training sets. Among them, 151 patients (30.82%) experienced recurrenced, including 58 (30.61%) in the validation set and 106 (30.90%) in the training sets. LASSO and MLR analyses identified migraine (OR = 2.208, 95% CI = 1.278–3.817), serum calcium (OR = 0.601, 95% CI = 0.447–0.81), 25(OH)D (OR = 0.785, 95% CI = 0.713–0.864), and serum estradiol (OR = 0.820, 95% CI = 0.752–0.894) as significant factors influencing recurrence within 1 year after CRP treatment in postmenopausal women with BPPV.

**Conclusion:**

The recurrence rate of BPPV within 1 year after CRP treatment in postmenopausal women is high. Migraine, 25(OH)D, calcium, and estradiol are associated with recurrence. The risk prediction model, developed using these factors, demonstrates good discrimination and calibration. It effectively predicts the recurrence risk within 1 year after successful CRP treatment, offering practical clinical value.

## 1 Introduction

Benign paroxysmal positional vertigo (BPPV) is the most prevalent vestibular disorder encountered in emergency departments, neurology units, and otolaryngology clinics (1). The lifetime prevalence of BPPV is approximately 10%, affecting over 420 million adults worldwide, making it a significant health concern (2, 3). While most BPPV patients recover following the Canalith Repositioning Procedure (CRP), some experience recurrences after treatment (4). Studies indicate that 15–30% of patients have a recurrence within the first year, and cumulative recurrence rates can reach to 50% over 10 years (5). Recurrent BPPV episodes lead to reduced productivity 68% of patients, job changes for 4%, and resignation for 6%. Additionally, these occurrences can trigger depression, anxiety, and other mental health issues (6). Therefore, identifying risk factors for BPPV recurrence is essential to reduce healthcare costs and alleviate patient suffering.

Epidemiological studies suggest that the incidence of BPPV in postmenopausal women is 2–3 times higher than in men, indicating a potential link between declining estrogen levels after menopause and the disorder (7). Otoliths, which are consisted of calcium carbonate crystals, and affected by abnormal calcium metabolism, a key factor in BPPV. Vitamin D play a role in regulating calcium and phosphate homeostasis, while estrogen is essential for bone formation, maintenance, and resorption. Thus, it is hypothesized that osteoporosis and abnormal calcium metabolism, resulting from hormonal changes in postmenopausal women, may contribute to BPPV recurrence (8). Several studies have demonstrated that estrogen-related calcium metabolism disorders and vitamin D deficiency as risk factors for BPPV recurrence in postmenopausal women. Nevertheless, other research argues that BPPV recurrence is not associated with vitamin D levels but to systemic conditions such as diabetes, hypertension, hyperlipidemia, migraines, cardiovascular diseases, and thyroid disorders (5). As such, there is no consensus on the risk factors for BPPV recurrence in postmenopausal women.

Risk prediction models are statistical tools that use multiple variables to predict outcomes, helping assess clinical scenarios such as disease occurrence, risk, and prognosis. These models allow physicians to better evaluate patients’ risks and prognoses. Postmenopausal women are a high-risk group for BPPV recurrence due to hormonal changes. Developing a risk nomogram prediction model (NPM) for recurrence can enable early identification and intervention for high-risk patients, thus preventing recurrence. This multicenter cross-sectional study used medical data to apply least absolute shrinkage and selection operator (LASSO) regression for variable selection, followed by multivariate logistic regression (MLR) analysis. A NPM was then developed to estimate BPPV recurrence within 1 year after successful repositioning treatment in postmenopausal women, offering a clinical reference for preventing recurrence in this population.

## 2 Materials and methods

### 2.1 Subjects

Postmenopausal female BPPV patients diagnosed and successfully treated at five hospitals in Sichuan Province (Affiliated Hospital of North Sichuan Medical College, Suining Central Hospital, Meishan City People’s Hospital, Nanbu County People’s Hospital) from January 2019 to January 2024 were selected for this study. Inclusion criteria included: postmenopausal women meeting China’s diagnostic criteria for BPPV; successfully treated with CRP; an education level of at least 6 years; signed informed consent; vertigo triggered by head position changes; typical positional nystagmus; absence of central nervous system diseases, vestibular dysfunction, thyroid diseases, gynecological diseases, or recent hormonal drug use; resolution of positional nystagmus after BPPV treatment; and no use of medications following after successful CRP treatment (1).

Exclusion criteria included: patients with incomplete recovery from craniocerebral injury; unresolved suppurative otitis media; central nervous system injuries or other factors affecting brain structure and function; chronic renal failure (eGFR <60 mL/min/1.73 m^2^ for ≥3 months); abnormal parathyroid hormone (PTH) levels (normal range: 15–65 pg./mL); mental illnesses or neurological conditions that could interfere with study participation; and individuals who had received osteoporosis treatment, calcium therapy, or vitamin D supplementation (2).

**Figure 1:**
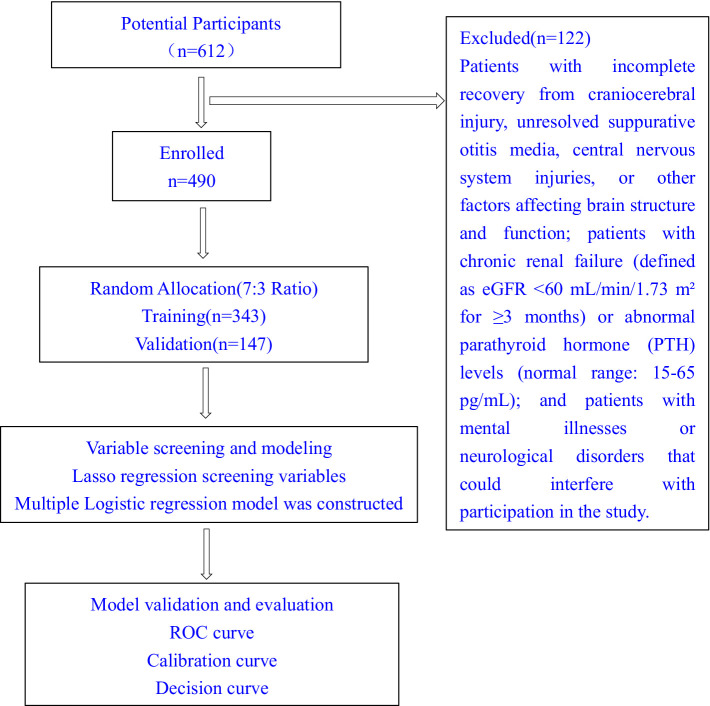
Study flowchart.

The sample size was determined based on: (1) The 10 events per predictor variable (EPV) rule for multivariable regression, requiring at least 10 events per predictor; (2) Initial candidate predictors (*n* = 19) were reduced to 6 variables through LASSO to meet 10 EPV requirement, ≥60 events were needed. With 151 recurrence events (exceeding the threshold) and a 20% dropout buffer, the final sample size was sea at 490.These 490 patients were randomly assigned to training (*n* = 343, 70%) and validation (*n* = 147, 30%) sets through stratified randomization. The study was approved by the AHNSMC Ethics Committee (Ethics number: 2020ER035-1), and all participants provided informed consent (Figure 1).

### 2.2 Test index

Data collected from patients included age, years of education, residence, body mass index (BMI), hypertension, hyperlipidemia, hyperglycemia, migraine, cervical spondylosis, Meniere’s disease, osteoporosis, history of head trauma, otolith site, Dizziness Handicap Inventory (DHI) score, Hospital Anxiety and Depression Scale (HADS) anxiety subscale (HADS-A), HADS Depression subscale (HADS-D), Pittsburgh Sleep Quality Index (PSQI), and levels of estradiol, calcium, and 25(OH)D.

#### 2.2.1 Hypertension

Defined as diastolic blood pressure ≥90 mmHg or systolic blood pressure ≥140 mmHg, according to the 2010 Hypertension Guidelines (9).

#### 2.2.2 Hyperlipidemia

Diagnosed when triglycerides and fasting serum total cholesterol levels were ≥1.7 mmol/L and ≥5.72 mmol/L, respectively (10).

#### 2.2.3 Hyperglycemia

Defined as blood glucose level of ≥11.1 mmol/L 2 hours post-meal and/or fasting blood glucose ≥7.0 mmol/L (11).

#### 2.2.4 Bone mineral density (BMD)

Measured using a Hologic whole-body dual-energy X-ray bone densitometer at the lumbar spine (L1-L4). The *T*-value is calculated as: *T*-value = (measured BMD- normal youth’s BMD reference)/normal youth’s BMD reference.

Based on *T*-values, patients were classified as having normal bone mass (*T*-value: > − 1.0), decreased bone mass (*T*-value: −1.0– −2.5), or osteoporosis (*T*-value: <−2.5) (12).

#### 2.2.5 25(OH)D, calcium, and estradiol levels

Blood samples were collected in the morning (08:00–10:00) to minimize diurnal variation in 25(OH)D levels. Patients enrolled from January 2019 to January 2024 were stratified by season (spring: March–May; summer: June–August; autumn: September–November; winter: December–February), with balanced recruitment across four calendar years to account for seasonal effects. After BPPV diagnosis and successful repositioning, venous blood was extracted, centrifuged for 15 min at 3000 rpm, and the supernatant was collected. 25(OH)D and estradiol concentrations were determined using an immunofluorescence analysis system, while calcium levels were measured with a semi-automatic analyzer. 25(OH)D levels were classified according to the Endocrine Society Clinical Practice Guidelines: deficiency (<20 ng/mL), insufficiency (20–30 ng/mL), and sufficiency (>30 ng/mL). Calcium levels were considered normal between 2.25 and 2.75 mmol/L, with values below 2.25 mmol/L classified as abnormal (13).

#### 2.2.6 DHI scale scoring

The DHI quantitatively evaluates dizziness severity. Score of 0–30, 31–60, and 61–100 indicate mild, moderate, and severe impairment, respectively (14).

#### 2.2.7 Hospital anxiety and depression scale (HADS)

This scale consists of 14 items, each scored from 0 to 3, with eight items requiring reverse scoring. It includes two subscales: HADS-D and HADS-A, each with a maximum score of 21. Scores are classified as follows: 0–7 points indicate normal levels, 8–10 points suggest slight abnormality, and scores ≥ 11 indicate abnormality. Patients with slightly abnormal or abnormal scores on the HADS-D and HADS-A are considered to have depression and anxiety, respectively (15).

#### 2.2.8 Pittsburgh sleep quality index (PSQI)

The PSQI assesses sleep quality with a maximum score of 21. Score of 0–7 indicate good sleep quality, while scores ≥8 suggest poor sleep quality (16).

### 2.3 Statistical analysis

Data conforming to or approximating a normal distribution were expressed as mean ± standard deviation (±SD), with independent sample *t*-test used to compare difference. For non-normally distributed data, the median was used and rank-sum tests were applied to assess differences. Categorical data were expressed as frequencies or percentages, and *χ*^2^ tests were utilized to evaluate group variances.

Participants were randomly separated into validation and training sets in a 3:7 ratio. LASSO regression was applied to screen independent variables, with penalty terms used to compress estimated parameters and identify significant factors influencing dependent variables (17). The selected variables were further assessed utilizing MLR to develop a risk NPM.

Model performance was assessed utilizing the area under the receiver operating characteristic (ROC) curve (AUC) to measure accuracy. Calibration was assessed with the Hosmer-Lemeshow calibration test and calibration curves to evaluate the model’s fit. A significance threshold of *α* = 0.05 was set, with *p* < 0.05 considered statistically significant.

## 3 Results

### 3.1 Patient data in training and validation sets

A total of 490 patients were enrolled and randomly separated into the validation (*n* = 147) and training (*n* = 343) sets in a 3:7 ratio. Of these, 30.61% (45/147) in the validation set and 30.90% (106/343) in the training set experienced recurrence, resulting in an overall recurrence rate was 30.82% (151/490). The median follow-up duration was 12 months (range: 10–14 months), with systematic follow-up assessments conducted at 1, 3, 6, and 12 months post-treatment to ensure consistency. Patients in both sets were evaluated based on age, place of residence, BMI, and the presence of hypertension, hyperlipidemia, hyperglycemia, migraine, cervical spondylosis, Meniere’s disease, and osteoporosis. As shown in Table 1, there were no statistically significant differences (*p* > 0.05) between the groups in terms of otolith site, DHI score, HADS-A, HADS-D, PSQI, VAS, estradiol, blood calcium concentration, or 25(OH)D concentration (Table 2).

**Table 1:** Demographic and clinical information in training set and validation set [±s or M (P25, P75)].

	Training set (*n* = 343)	Validation set (*n* = 147)	*χ*^2^/t/z	*p*
Age (years)	59.13 ± 6.17	59.03 ± 4.79	0.165	0.869
Place of abode (%)			2.046	0.153
Country	99 (28.86)	52 (35.37)		
City	244 (71.14)	95 (64.63)		
BMI	27.17 ± 2.64	27.37 ± 2.59	−0.769	0.443
Hypertension (%)			0.277	0.599
NO	244 (71.14)	108 (73.47)		
YES	99 (28.86)	39 (26.53)		
Hyperlipidemia (%)			1.006	0.316
NO	262 (76.38)	106 (72.11)		
YES	81 (23.62)	41 (27.89)		
Diabetes (%)			0.009	0.923
NO	272 (79.3)	116 (78.91)		
YES	71 (20.7)	31 (21.09)		
Migraine (%)			1.767	0.184
NO	219 (63.85)	103 (70.07)		
YES	124 (36.15)	44 (29.93)		
Cervical spondylosis (%)			0.931	0.335
NO	279 (81.34)	114 (77.55)		
YES	64 (18.66)	33 (22.45)		
Meniere disease (%)			1.36	0.237
NO	271 (79.01)	109 (74.15)		
YES	72 (20.99)	38 (25.85)		
Osteoporosis (%)			0.686	0.407
NO	228 (66.47)	92 (62.59)		
YES	115 (33.53)	55 (37.41)		
The site of the otolith (%)			2.829	0.419
Posterior semicircular canal	142 (41.4)	52 (35.37)		
Horizontal semicircular canal	89 (25.95)	47 (31.97)		
Anterior semicircular canal	63 (18.37)	30 (20.41)		
Mixed type	49 (14.29)	18 (12.24)		
DHI score (%)			2.567	0.277
Mild	136 (39.65)	67 (45.58)		
Moderate	138 (40.23)	48 (32.65)		
Severe	69 (20.12)	32 (21.77)		
HADS-A scores			1.795	0.18
Normal	246 (71.72)	114 (77.55)		
Abnormal	97 (28.28)	33 (22.45)		
HADS-D scores			0.054	0.816
Normal	283 (82.51)	120 (81.63)		
Abnormal	60 (17.49)	27 (18.37)		
PSQI scores			0.176	0.675
Normal	265 (77.26)	111 (75.51)		
Abnormal	78 (22.74)	36 (24.49)		
VAS scores	5 (4.6)	5 (4.6)	−0.172	0.863
Serum calcium (mmol/L)	2.25 ± 1.00	2.31 ± 0.88	−0.559	0.577
25(OH)D (ng/ml)	19.16 ± 3.14	19.29 ± 3.05	−0.419	0.675
Serum estradiol (ng/l)	16.49 ± 3.30	16.24 ± 2.81	0.794	0.428

BMI, body mass index; DHI, Dizziness Handicap Inventory; HADS, Hospital Anxiety and Depression Scale; HADS-A, Anxiety subscale; HADS-D, Depression subscale; PSQI, Pittsburgh Sleep Quality Index. All *p*-values represent between-group comparisons between the training set (*n* = 343) and validation set (*n*, 147). For continuous variables, *t*-tests were used for normally distributed data (e.g., age, BMI), while Mann–Whitney U tests were applied to non-normally distributed data (e.g., VAS scores). Categorical variables were analyzed using χ^2^ tests (expected frequency ≥5) or Fisher’s exact tests (expected frequency <5).

**Table 2:** Assignment methods of argument variables.

Variable	Assignment mode
Migraine	NO = 0; YES = 1
Osteoporosis	NO = 0; YES = 1
HADS-A	Normal = 0; Abnormal = 1
Calcium	Continuous variable
25(OH)D	Continuous variable
Serum estradiol	Continuous variable

### 3.2 Univariate analyses of risk factors

**Figure 2:**
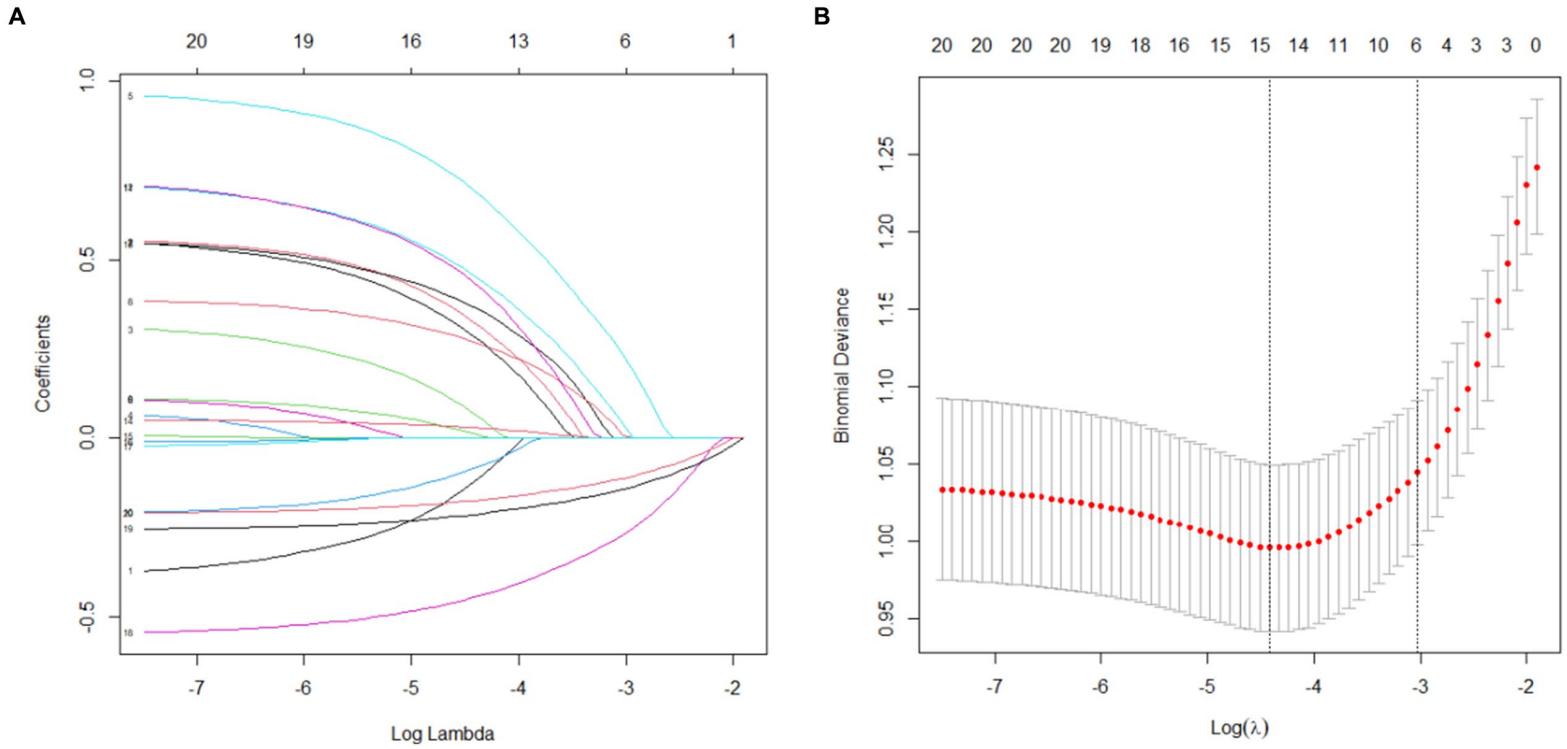
LASSO regression results **(A)** Variable selection; **(B)** Cross-validation.

LASSO regression was utilized to identify predictors with non-zero coefficients from 19 variables. Using 10-fold cross-validation, the optimal *λ* value was determined to include the fewest variables while maintaining model fit. Ultimately, six predictors with non-zero coefficients were selected: migraine, osteoporosis, HADS-A, calcium, 25(OH)D, and serum estradiol, as shown in Figure 2.

### 3.3 Multivariate analysis of risk factors

A MLR model was established with recurrence as the dependent variable, and LASSO regression was utilized to select six variables as independent variables. The multivariate analysis identified migraine (OR = 2.208), serum calcium (OR = 0.601), 25(OH)D (OR = 0.785), and estradiol (OR = 0.820) as independent predictors of recurrence (all *p* < 0.05), as detailed in Table 3.

**Table 3:** Final predictors in multivariable logistic regression model.

	B	S.E.	Wald	*p*	OR	95% CI
Migraine	0.792	0.279	8.052	0.005	2.208	1.278—3.817
Serum calcium	−0.509	0.152	11.239	0.001	0.601	0.447—0.81
25(OH)D	−0.242	0.049	24.458	<0.001	0.785	0.713—0.864
Serum estradiol	−0.199	0.044	20.274	<0.001	0.820	0.752—0.894

Among 6 variables initially selected by LASSO regression, only 4 retained statistical significance (*p* < 0.05).

### 3.4 Nomogram construction and predictive performance

**Figure 3:**
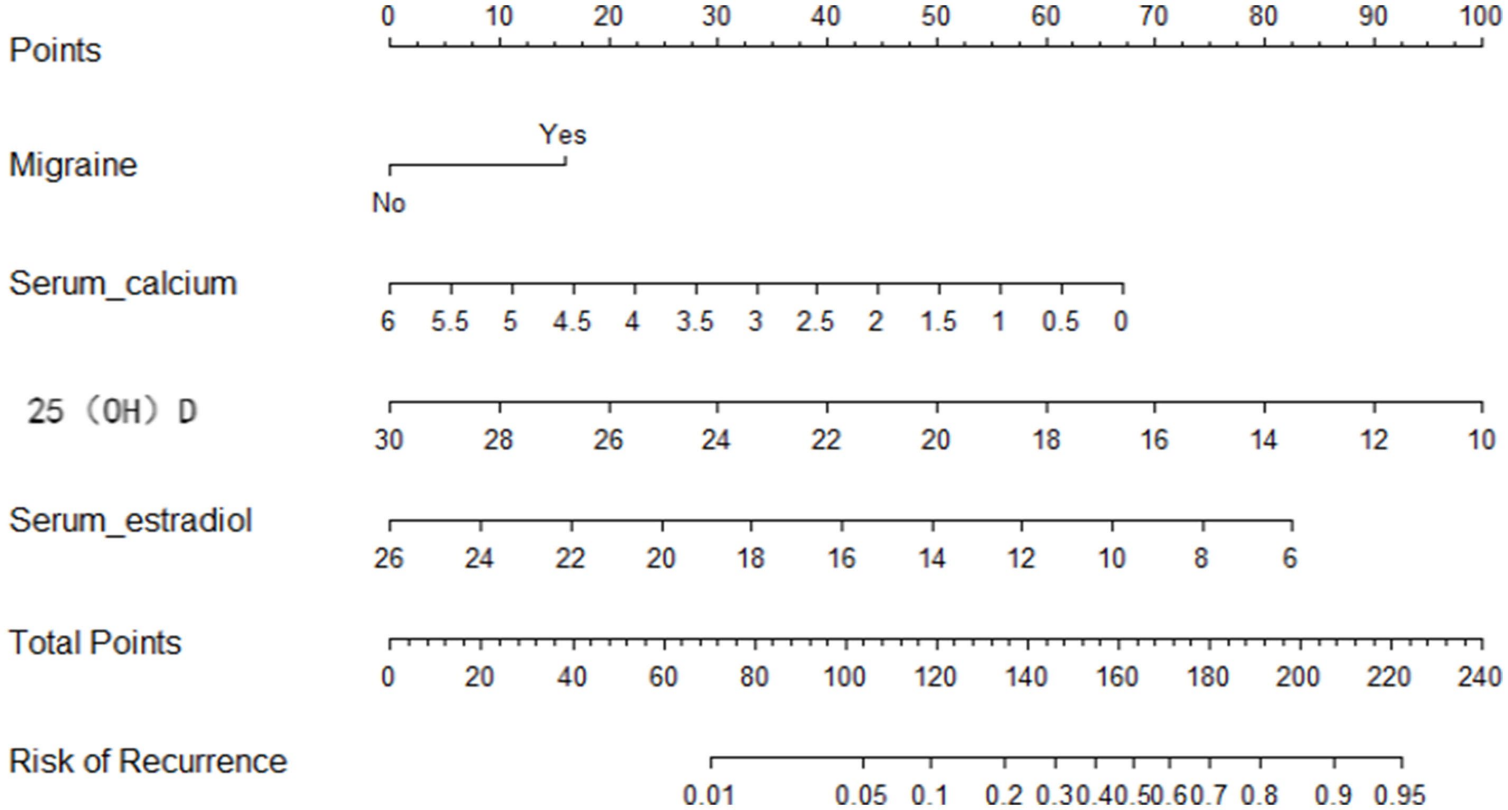
Nomogram prediction model.

A NPM was constructed based on the MLR analysis to estimate the recurrence risk in postmenopausal women with BPPV. The model included migraine, estradiol, calcium, and 25(OH)D, as shown in Figure 3. For example, consider a 58-year-old postmenopausal women with BPPV successfully treated with CRP. Her medical history includes migraine (Yes); serum calcium: 2.0 mmol/L (below normal range: 2.25–2.75 mmol/L); 25(OH)D level: 18 ng/mL (deficiency: <20 ng/mL); Serum estradiol: 15 ng/L (postmenopausal normal range: typically <30 ng/L). Based on the nomogram (Figure 3), the points for each variable are assigned as follows (hypothetical values; actual points depend on nomogram scales): Total Points = 30 (Migraine (Yes)) + 45 (Serum calcium (2.0 mmol/L)) + 50 (25(OH)D (18 ng/mL)) + 40 (Estradiol (15 ng/L)) = 165 points. According to the nomogram’s risk axis, 165 points correspond to a 65% 1-year recurrence probability (calibration based on model-specific curves). This patient is at high risk (>50% recurrence).

**Figure 4:**
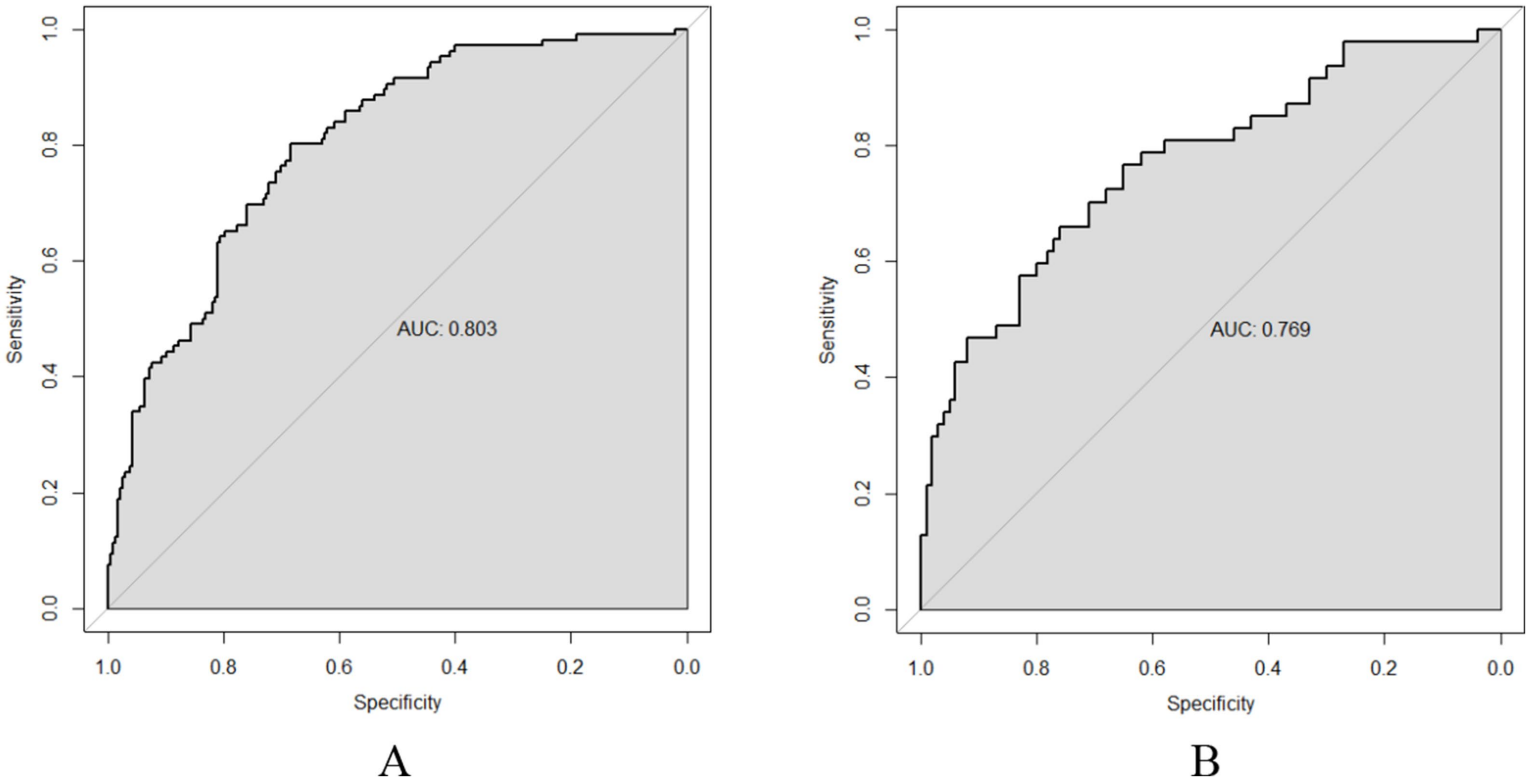
ROC curves **(A)** Training set; **(B)** Validation set.

**Figure 5:**
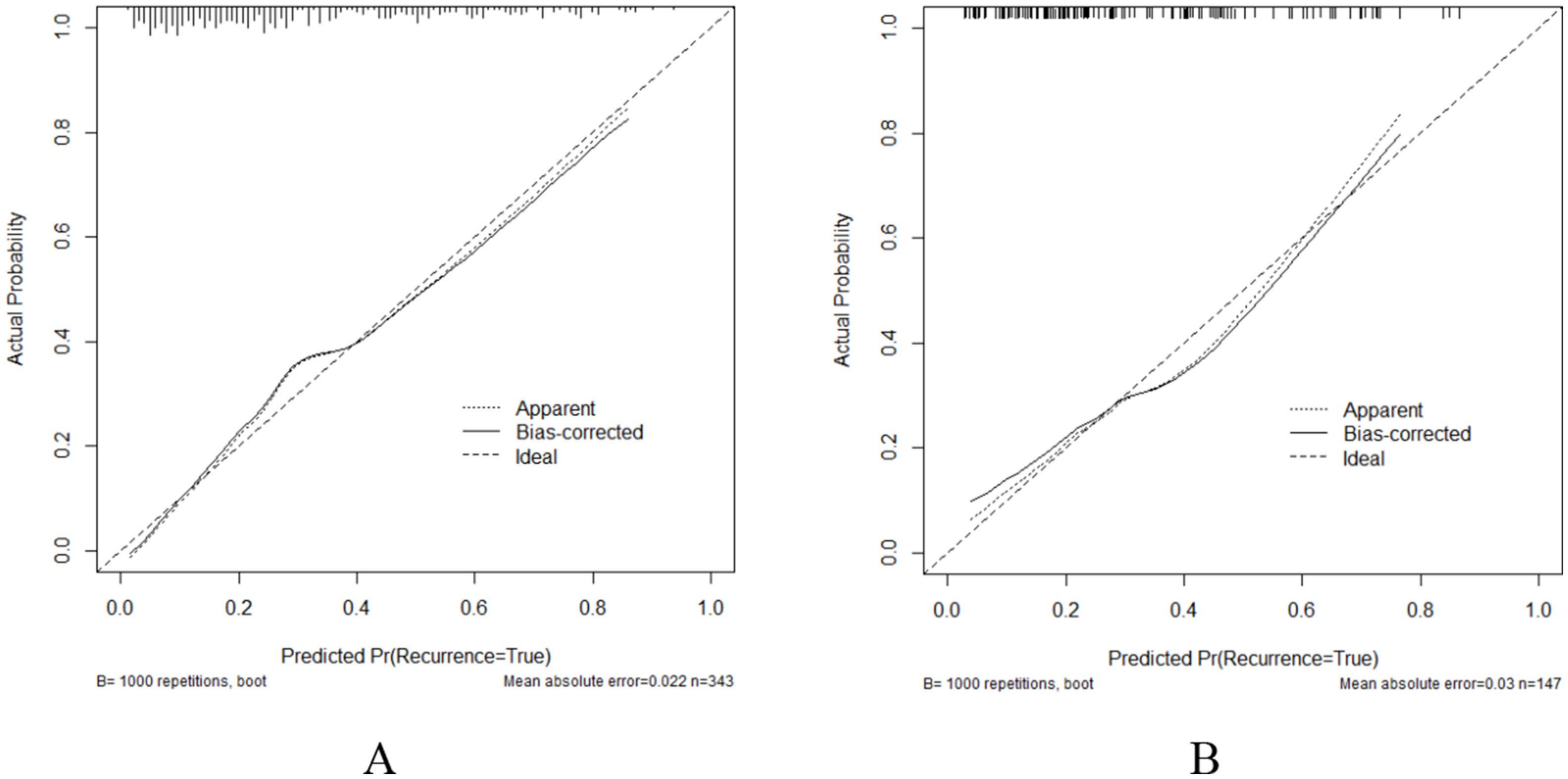
Calibration curves **(A)** Training set; **(B)** Validation set.

The ROC curve was utilized to assess the model’s ability to differentiate recurrence. The AUCs for the validation and training sets were 0.769 (95% CI: 0.6855–0.852; Figure 4B) and 0.803 (95% CI: 0.7555–0.851; Figure 4A), respectively. The HLC test yielded chi-square values of 8.836 (*p* = 0.356) and 7.930 (*p* = 0.440) for the validation and training sets, respectively, indicating a good fit for both sets (Figure 5). These results show no significant differences between the calibration curve and the ideal NPM curve, demonstrating alignment between predicted and actual values.

**Figure 6:**
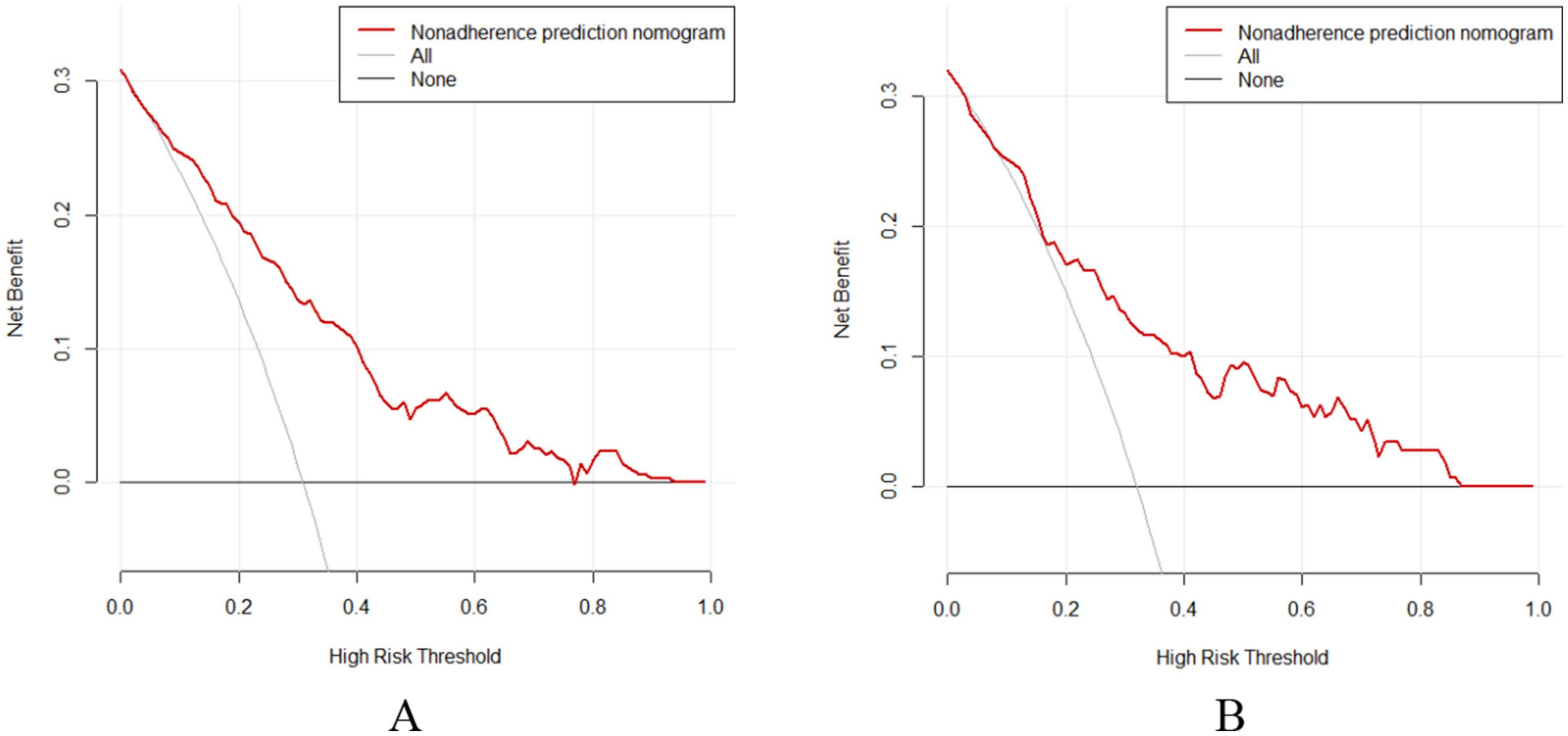
DCA curves **(A)** Training set; **(B)** Validation set.

The DCA showed that when the probability thresholds for the validation and training sets were 0.160–0.860 and 0.050–0.760 (Figure 6), both were above the “None” and “All” lines. Therefore, using this prediction model for recurrence in postmenopausal women with BPPV offers more clinical value when early intervention is provided to patients at risk within this range, compared to treating all patients.

## 4 Discussion

A total of 490 patients were enrolled, with 147 in the validation set and 343 in the training sets. Among them, 30.61% (45/147) in the validation set and 30.90% (106/343) in the training set experienced recurrence, resulting in an overall recurrence rate was 30.82% (151/490). LASSO regression and MLR analyses identified migraine, 25(OH)D, calcium, and estradiol as independent risk factors for recurrence in postmenopausal women with BPPV following successful treatment. 25(OH)D Deficiency demonstrated the strongest association with recurrence. For every 10 ng/mL decrease in 25(OH)D, the recurrence risk increased by 2.15-fold. Hypoestrogenemia was the second strongest effect, with women in the lowest estradiol quartile (<13.2 ng/L) having 3.08-fold higher risk than those in the highest quartile (>19.1 ng/L). Migraine History, while having the highest odds ratio, had a moderately lower Wald statistic, suggesting a substantial effect but with slightly wider confidence intervals. Hypocalcemia showed a paradoxical ranking - while statistically significant, its clinical relevance was limited by the narrow physiological range (2.1–2.6 mmol/L) in our cohort. This hierarchy suggests that chronic metabolic derangements, such as vitamin D and estrogen deficiency, may have a greater cumulative biological impact on recurrence than episodic neurological factors like migraine.

Migraine is a significant risk factor for the recurrence of BPPV in postmenopausal women. It is a complex neurological disorder that affects 18% of women and 6% of men globally, typically characterized by headaches accompanied by reversible visual, sensory, and/or motor symptoms. Research indicates that the incidence of BPPV in migraine patients is 2 times higher than in individuals without migraine (18). Calhou’s study of 425 migraine patients found a significant association between migraine severity and vertigo symptoms (19). Faralli et al. also demonstrated that BPPV patients with a history of migraine have an extremely high recurrence rate (20). Both migraine and BPPV can present with vertigo, making it challenging to distinguish between the two. The recognized pathogenesis of BPPV involves clinical symptoms and signs of disturbed endolymphatic flow caused by the displacement of otolith particles into the semicircular canal, leading to the deviation of the ampullary crest (1). The theory of vasospasm, related to migraine pathogenesis, may help explain the occurrence of BPPV. Vasospasm or vestibulo-microvascular disorders could result in inner ear ischemia, triggering the loss of otoliths from the utricular macula, leading to BPPV (21). This study shows that postmenopausal women with both migraine and BPPV have a higher risk of recurrence. Therefore, in clinical practice, it is essential to thoroughly investigate the history of migraine in BPPV patients and conduct more detailed migraine-related examinations to better understand and manage the risk of recurrence.

The recurrence of BPPV in postmenopausal women is closely associated with calcium levels. Postmenopausal women are more prone to osteoporosis and metabolic bone diseases due to hormonal changes, which may affect calcium ion concentration in the inner ear lymph fluid and lead to otolith dissolution. It is clear that bone turnover markers, such as blood calcium levels, play a role in the pathogenesis of BPPV. Otoliths, which are formed by the crystallization of calcium carbonate, are continuously mineralized by calcium ions, a process influenced by the calcium ion concentration in the inner ear lymph fluid (22). After maturity, otoliths undergo dynamic turnover, constantly being worn down and replenished (23). The outer lymph of the inner ear, a filtrated of blood, forms endolymph fluid through filtration (24), which means blood calcium concentration can influence the occurrence and recurrence of BPPV by affecting the endolymphatic Ca^2+^ concentration. Hypocalcemia (<2.25 mmol/L) may impair otoconial stability through three key pathways: (1) altered calcium gradients reduce mineralization efficiency of matrix proteins like otoconin-90; (2) endolymphatic calcium dyshomeostasis triggers abnormal shedding of immature otoconia; (3) dysregulated calcium-sensing receptor (CaSR) signaling disrupts mechanotransduction in vestibular hair cells (24). Our study further identified decreased serum calcium levels as an independent risk factor for BPPV recurrence This finding aligns with the pathophysiological mechanisms of calcium metabolism in the inner ear. Notably, in postmenopausal women, estrogen deficiency-induced bone resorption creates a ‘calcium paradox’—while transient elevation of serum calcium occurs through bone mobilization, chronic vitamin D deficiency ultimately disrupts calcium homeostasis. This underscores the clinical necessity for comprehensive monitoring of the calcium-vitamin D-estrogen axis.

Kahraman et al. found that 93.5% of BPPV patients had reduced vitamin D levels (25). Similarly, Jeong et al. also found that 80% of BPPV patients had vitamin D levels below 20 ng/mL (26). Talaat et al. observed a significantly higher recurrence rate in patients with severe vitamin D deficiency (27). Buki et al. found that no BPPV patients experienced recurrence after more than 8 months of follow-up with vitamin D supplementation (28). Vitamin D regulates calcium metabolism and promotes calcium absorption in the small intestine, which is crucial for bone mineralization (29). Additionally, vitamin D affects the expression of calcium-binding proteins and calcium ion channels in inner ear epithelial cells, with serum 25(OH)D being one of best indicators of vitamin D status (30). To minimize confounding effects on calcium and vitamin D metabolism, we excluded patients with renal dysfunction and abnormal PTH. This study suggests that 25(OH)D deficiency is a risk factor for BPPV recurrence after menopause. Vitamin D deficiency may lead to BBPV occurrence, recurrence, and persistence through impaired calcium metabolism in the inner ear. Recent research has shown that vitamin D supplementation reduces the recurrence rate of BPPV (31). Although 25(OH)D levels are influenced by season and circadian rhythms, our standardized morning sampling protocol and multi-year stratified recruitment effectively controlled these confounders, improving the validity of the findings. Future studies will explore different interventions for recurrence risk factors in postmenopausal women with BPPV and assess how these factors influence the effectiveness of postoperative CRP treatment to reduce recurrence.

The recurrence of BPPV in postmenopausal women is closely related to estradiol concentration. Estrogen, a glucocorticoid with 18 carbon atoms, is synthesized from androgens by aromatase, primarily in the forms of estradiol, estrone, and estriol (32). Estrodiol, the most active form of estrogen, influences various biological systems, including the reproductive, cardiovascular, and central nervous systems, as well as pancreatic function and bone metabolism, by binding to estrogen receptors (33). Before menopause, estrogen is predominantly produced by the ovaries, but after menopause, estrogen secretion declines sharply. Estrogen’s involvement in BPPV pathogenesis may occur through several mechanisms: First, estrogen binds to receptors in bone and bone marrow, participating in bone metabolism. A decrease in estrogen levels can increase bone resorption, reduce bone density, alter the composition of otoliths, and cause them to calcify and fall off, leading to BPPV (34). Second, estrogen promotes calcitonin secretion and influences calcium metabolism. A reduction in estrogen levels leads to decreased calcitonin secretion, disrupting calcium metabolism and increasing free calcium ions in the inner ear lymph fluid, impairing otolith degradation and affecting otolith formation and shedding (35). Third, estrogen receptors in the inner ear regulate inner ear microcirculation. Estrogen deficiency can disturb inner ear circulation, leading to otolith detachment and BPPV (23). Yang et al. found that postmenopausal women with BPPV had significantly lower estrogen levels compared to controls, suggesting estrogen may influence BPPV occurrence by regulating otoconia-90 protein expression (36). In a rat model of menopause with bilateral ovariectomy, estrogen replacement therapy reversed the deterioration in otoconia-90 protein levels (37). This study indicates that lower estrogen levels are a significant risk factor for BPPV recurrence in postmenopausal women. However, most studies on estrogen and BPPV are longitudinal, and future prospective cohort studies are need to be needed to validate the association between BPPV and estrogen levels in postmenopausal women.

### 4.1 Limitations

This study used LASSO regression for variable screening and logistic regression to construct a risk nomogram prediction model for postmenopausal women with BPPV after successful reduction. The model demonstrated good differentiation and calibration in both the validation and training sets. However, the recurrence period in this study was limited to 1 year, which is relatively short. Future studies could extend the observation period to gather more comprehensive recurrence data. Furthermore, this study did not conduct a prospective cohort investigation on risk factors, and future research should focus on developing prospective interventions to prevent BPPV recurrence in postmenopausal women. Fourth, while semicircular canal localization data were collected, the study did not analyze subtype-specific recurrence patterns due to sample size constraints. Future large-scale studies should investigate whether metabolic risk factors (e.g., vitamin D deficiency, hypoestrogenemia) exhibit differential effects across posterior, horizontal, and anterior canal BPPV subtypes.

## 5 Conclusion

In conclusion, the nomogram prediction model constructed in this study, based on migraine, 25(OH)D, estradiol, and calcium, is simple, easy to use, and demonstrates good accuracy and prediction efficiency. This model can effectively identify postmenopausal women at high risk of BPPV recurrence after successful reduction, offering valuable insights for formulating preventive measures to reduce recurrence in this population.

## Data Availability

The raw data supporting the conclusions of this article will be made available by the authors, without undue reservation.
